# The Brazilian primary health care response to the COVID-19 pandemic: individual and collective approaches

**DOI:** 10.3389/fpubh.2023.1212584

**Published:** 2023-12-08

**Authors:** Aylene Bousquat, Ligia Giovanella, Luiz Facchini, Maria Helena Magalhaes Mendonça, Fulvio Borges Nedel, Geraldo Cury, Paulo Henrique dos Santos Mota, Simone Schenkman, Patricia Sampaio Chueiri, Maria Cecilia Goi Porto Alves

**Affiliations:** ^1^Department of Politics, Management and Health, Faculty of Public Health, University of São Paulo, São Paulo, Brazil; ^2^National School of Public Health (ENSP), Rio de Janeiro, Brazil; ^3^Department of Social Medicine, Faculty of Medicine, Federal University of Pelotas, Pelotas, Brazil; ^4^Department of Public Health, Federal University of Santa Catarina, Florianopolis, Brazil; ^5^Department of Social and Preventive Medicine, Faculty of Medicine, Federal University of Minas Gerais, Belo Horizonte, Brazil; ^6^Faculdade Israelita de Ciências da Saúde Albert Einstein Hospital Israelita Albert Einstein, São Paulo, Brazil; ^7^Department of Health, Institute of Health, Government of the State of São Paulo, Institute of Health, São Paulo, Brazil

**Keywords:** primary health care, COVID-19 pandemic, Brazil, health services, family health, health survey

## Abstract

**Objectives:**

Brazil’s PHC wide coverage has a potential role in the fight against COVID, especially in less developed regions. PHC should deal with COVID-19 treatment; health surveillance; continuity of care; and social support. This article aims to analyze PHC performance profiles during the pandemic, in these axes, comparing the five Brazilian macro-regions.

**Methods:**

A cross-sectional survey study was carried out, using stratified probability sampling of PHC facilities (PHCF). A Composite Index was created, the Covid PHC Index (CPI). Factor analysis revealed that collective actions contrastingly behaved to individual actions. We verified differences in the distributions of CPI components between macro-regions and their associations with structural indicators.

**Results:**

Nine hundred and seven PHCF participated in the survey. The CPI and its axes did not exceed 70, with the highest value in surveillance (70) and the lowest in social support (59). The Individual dimension scored higher in the South, whereas the Collective dimension scored higher in the Northeast region. PHCF with the highest CPI belong to municipalities with lower HDI, GDP *per capita*, population, number of hospitals, and ICU beds.

**Conclusion:**

The observed profiles, individually and collectively-oriented, convey disputes on Brazilian health policies since 2016, and regional structural inequalities.

## Introduction

The fight against the coronavirus disease (COVID-19) pandemic has been a task of enormous magnitude for all health systems, requiring a broad and articulated set of responses. Unfortunately, these were not observed in most countries at the beginning of 2020, which contributed to the high rates of morbidity and mortality (1). At first, the actions were almost exclusively directed to capacity expansion of hospital and intensive care beds. However, it quickly became clear that these actions would be insufficient to overcome the pandemic (2, 3). Robust intelligence systems with notifying actions; well-defined PHC services; social isolation measures and social support policies for vulnerable populations; strong health surveillance associated with community actions; and significant investments for vaccine development, were pointed out as essential to face the most dramatic health situation in the last 100 years (4, 5).

The first COVID-19 case was reported in Brazil on February 26, 2020, and on March 22 of the same year, reported cases were present in all Brazilian states. The pandemic led to a high number of deaths and cases, amounting to more than 600,000 deaths at the beginning of 2022, affecting especially the vulnerable population (6–9). The rates were higher and more accelerated in states with greater social inequality, such as in the black population, those with lower education levels and in the lowest income quintile, or living in the poorest city areas (10, 11). The dramatic scenario was partially relieved by the decentralized structure of the Brazilian health system, by which most state and municipal governments implemented actions to circumvent the obstacles imposed by the federal administration (12–15).

The Brazilian Unified Health System, called SUS (the acronym in Portuguese of *Sistema Único de Saúde*) has offered free and universal health care since 1990 (16). Despite its reduced funding, the SUS has improved the population’s health conditions and ensured the expansion of access to health services in general and particularly to primary health care (PHC). There are currently over 38,000 PHC facilities (PHCF) with strong capillarity throughout the territory (2020), which could play a central role in the fight against the COVID-19 pandemic. PHC strategies should be implemented in epidemic prevention, care, and control (17–20), especially when part of a global plan to face health emergencies, with better responses in some countries (21).

The Brazilian PHC model until 2017, has been the Family Health Strategy (FHS), characterized by the combination of individual care with strong community and regional actions. Its coverage comprises over 130 million people (63% of the population). Currently, about 43,000 FHS multi-professional teams are the system’s gateway and source of continued care for defined populations in specific territories (2020). It integrates health promotion, disease prevention, surveillance, treatment, and rehabilitation, delivered by physicians, nurses, dentists, and more than 300,000 Community Health Workers-CHW. This combination of Primary Care and essential public health functions (22) may have played an important role in preventing more critical outcomes in Brazil.

Medina et al. (19), proposed four essential axes for the Brazilian PHC organization model’s performance during the pandemic: COVID-19 treatment; health surveillance; continuity of care; and social support. The authors point out that conducting activities in these axes would allow a better fight against the pandemic and reduce the impact of COVID-19 on other population health needs.

This article, therefore, aims to analyze the performance profiles of PHC services during the pandemic and to compare the role of these four axes in the five Brazilian macro-regions, focusing on how PHC services reorganized themselves to face the COVID-19 pandemic.

## Methods

A cross-sectional survey study was carried out, using stratified probability sampling. The reference population comprised the set of PHCF registered in the National Registry of Health Establishments (CNES, *Cadastro Nacional dos Estabelecimentos de Saúde*) in December 2020. This population was stratified considering the five Brazilian macro-regions. For the smallest (North/Midwest), intermediate (South), and largest (Southeast/Northeast) ones, the sample sizes were defined as 100, 150, and 200, respectively, corresponding to sampling errors of 10, of 8 and 7 percentage points, totaling 750 units. For the country, the sampling error is 3.92, considering a design effect of 1.20, due to weighting.

Stratified sampling was employed and in each region the sample size was determined by the formula *𝑛= 𝑃 (1−𝑃)/(d/𝑧)^2^*, according to the population proportions estimates (*p* = 0.5); the sampling error tolerated (d) and the normal curve value (z = 1.96), corresponding to 95% confidence intervals.

Brazil has 33,495 Primary Healthcare facilities, distributed differently in the regions, along with the different social, economic, and demographic settings involved. The North(N) region has 2,717; the Northeastern (NE) has 13,393; the Midwest (MW) has 2,587; the Southeast (SE) has 10,189 and the South (S) region has 4,609 facilities. Thus, applying the formula above, and considering additional reserve units, we end up with 131 (N), 264 (NE), 132 (MW), 260 (SE), and 200 (S) facilities for the respective macro-regions.

The choice of analyzing the five macro-regions of Brazil stems from its heterogeneous territory, which lies in a pattern of socio-spatial exclusion, with important effects on the provision of public goods and services, including health, as shown in Table 1.

**Table 1:** Population, economic and health information. Brazil and Regions (2019).

	North	Northeast	Southeast	South	Midwest	Brazil
Population^a^	18,430,980	57,071,654	88,371,433	29,975,984	16,297,074	210,147,125
Population density^b^	4.8	36.7	95.6	53.2	10.1	24.7
Gross Domestic Product (GDP) *per capita*^b^ (R$)	22,811	18,359	44,330	42,438	44,876	35,162
Gini Index^b^	0.537	0.560	0.528	0.467	0.506	0.544
Percentage of population with adequate water supply^b^	90.5	91.4	99.6	99.5	99.3	96.5
Percentage of population with adequate sanitary facilities^b^	56.4	65.6	93.3	88.6	72.6	80.3
Percentage of population with regular waste collection/disposal^b^	71.7	74.8	91.9	90.5	86.4	84.9
Nurses SUS/1000 inhabitants^c^	0.9	1.1	1.1	1.2	1.1	1.1
Physicians SUS/1000 inhabitants^c^	0.9	1.1	1.7	1.7	1.5	1.4
Hospital beds SUS/1000 inhabitants^c^	0.8	1,0	0.7	1.1	0.9	0.9
UCI beds Adult SUS/100 k inhabitants^c^	4.6	5.9	8.1	9.2	6.8	7.2
UCI beds Neonatal SUS/1000 live births^c^	1.1	1.3	2,0	2.4	1.4	1.7
Ventilators in use SUS/100 k inhabitants^c^	14.2	16.2	23.8	22.6	23.5	20.7
% of hospital admissions sensitive to primary care^c^	28.7	24.5	20,0	20.9	21.9	22.1
Crude hospital admission rates (SUS)/1,000 inhabitants^c^	55.5	57.8	54.7	72.9	58.8	58.5
Infant mortality rates (< 1 year of age)/1,000 live births^c^	15.1	13.7	11.5	10.2	11.8	12.4
% of children under 1 year of age with immunization with tetravalent/pentavalent vaccine^c^	70.0	71.9	69.8	73.9	70.1	71.0
Estimated population coverage by Primary Health Care teams^c^	70.7	83.8	68.1	77.7	70.5	74.2
Population coverage (%) by private health plans/private insurance^d^	9.3	11.6	32.7	23.1	19.9	22.5

^a^Federal Accounting Court TCU.^b^Brazilian Institute of Geography and Statistics (IBGE).^c^DATASUS - SUS Information Technology Department SUS–Brazil’s Unified Health System.^d^National Supplementary Health Agency (ANS). Brazil and Regions (2019).

Considering a response rate of 80%, 945 PHCF were randomly sampled. The inclusion criteria for PHCF were: being operational during the pandemic period and having a doctor, nurse, or dentist working there for more than 6 months. Anticipating the need to exclude units after the beginning of the fieldwork, a reserve of units was also drawn aiming to replace those that did not belong to the study population (units mistakenly registered in the CNES as being Primary Care units) or that did not meet the inclusion criteria. A total of 40 units were included from this reserve. We had a very good response rate (95,8%), ranging from 90%(NE) to 98%(N; S; SE). We also had three rounds of substitutions for using the reserve sample (*n* = 40), according to the regional response rates (13 for the NE). This reserve was planned in case the PHCF was closed or did not meet the inclusion criteria, such as being another kind of health facility or not having a health professional as manager for more than 6 months.

**Figure 1:**
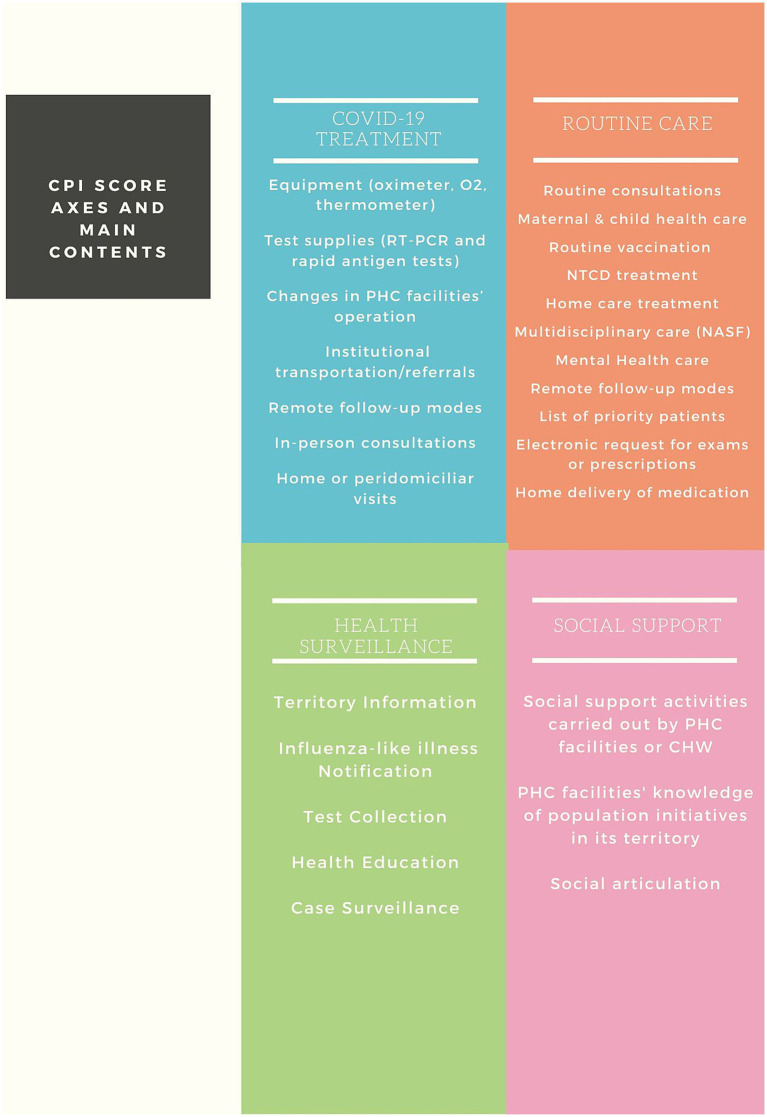
CPI blocs and aggregate variables.

The four axes of PHC action in the fight against COVID-19 proposed by Medina et al. (19) were used as a theoretical reference, guiding the creation of the questionnaire and the analysis of the results (Figure 1). Data collection was carried out between July 15 and November 12, 2021. In each selected PHCF, the manager or another health professional who met the inclusion criteria was invited to answer the online questionnaire. The study data were collected and managed using the Research Electronic Data Capture (REDCap) tool (23). This is a secure web-based software platform designed to support data capture for research studies. Among others, data were collected on: the PHCF’s physical structure and connectivity resources; basic supplies for COVID-19 treatment and its reorganization process; routine care; use of teleconsultation, telehealth, and telemonitoring; characteristics of access to the secondary and tertiary network in cases that required intensive clinical care; social support and surveillance actions in the territories.

The descriptive analysis consisted of characterizing the study population regarding the different variables gathered in the survey through the estimation of proportions and respective confidence intervals (95% confidence level), for each of the regions and the country as a whole.

The different probabilities of drawing samples in the strata for the selection of the units were compensated by the introduction of weights in the data analysis stage, corresponding to the inverse of the sampling fractions used in the strata.

After the descriptive analysis of the variables under the theoretical framework, we classified the PHCF according to the comprehensiveness of the actions carried out in the four axes, i.e., to identify which PHCF performed a greater number of actions considered crucial in the fight against COVID-19 in Brazil. For this purpose, a Composite Index was created, called the Covid Primary Health Care Index (CPI).

**CHART 1:**
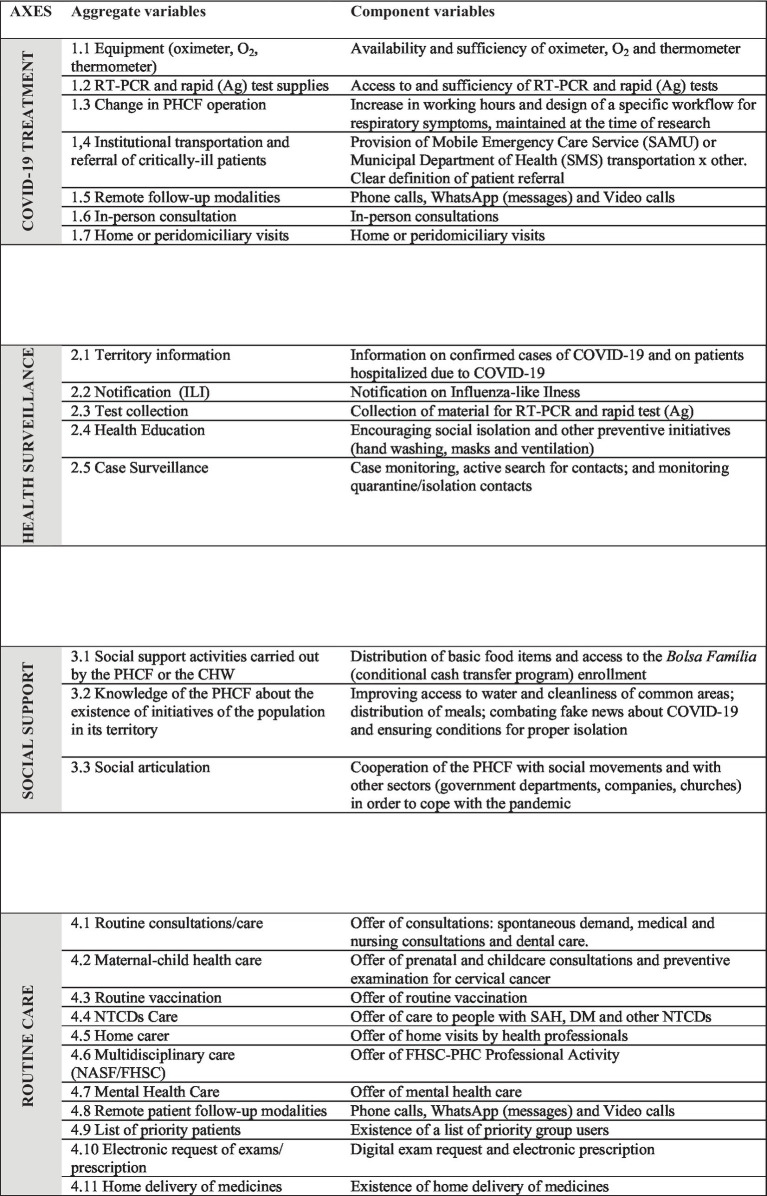
CPI axes: selected aggregate and component variables.

The process of creating the CPI started with the definition of the most relevant issues in each axis, which was carried out by a research panel, with experts on the Brazilian PHC. In this first stage, 59 questions were selected and aggregated into 26 variables distributed in the four axes (Chart 1). To verify and guarantee the coherence and consistency of the Index, the non-parametric correlations (Spearman) between the index, the axes, and the proposed variables were tested, followed by factor analysis (principal components analysis), aiming to validate its structure and, finally, consistency analysis (Cronbach’s alpha), leading to the final model. The details of this process can be seen in Supplementary material S1.

The index was built with equal weights for the axes and the variables in it, according to the formula below:

Covid Primary Health Care Index(CPI):


$$\mathrm{Index}={\bar{X}}_{e_{1}}^{e_{n}};\mathrm{where}\;e_{*}={\bar{X}}_{v_{1}}^{v_{n}}$$

Axes: e_1_ to e_n_ 0 ≤ e* ≤ 1.

Variables: v_1_ to v_n_ 0 ≤ v* ≤ 1.

In brief, a CPI with a value equal to 100 would represent a more complete performance pattern of the PHCF, and a value equal to zero, the failure to carry out any of the relevant actions. The same reasoning is valid for the respective score of each axis. To facilitate the discussion, the range from zero to one hundred was divided into quartiles, corresponding to very low, low, medium, and high values.

The different performance profiles were meant to assess mainly PHC service delivery, revolving around the inputs and outputs of this level. It’s important to emphasize that the CPI was not supposed to assess health levels or system outcomes. The survey was not designed in that manner and the five regions had different timeframes for the COVID-19 waves.

The factor analysis revealed that the axes that encompass more collective actions (Health Surveillance and Social Support) behaved similarly and in contrast to the axes more focused on individual actions (COVID-19 Treatment and Routine Care). Therefore, two dimensions were defined, the individual and the collective one, which articulate the axes and also express the characteristics of the Brazilian PHC.

### Data analysis

In order to verify possible differences in the distributions of the CPI, its dimensions, and variables between the regions, we used the median and interquartile range as references. We performed Kruskal-Wallis test for the set of data overall and Dunn’s test, with Bonferroni correction, for multiple comparisons between the regions.

We tested the differences in relative frequencies of respondents/PHCF characteristics between regions with the Chi-square test (Rao and Scott correction for complex samples).

We also verified associations between the CPI, categorized according to its median and the socioeconomic, demographic, political, structural, and COVID-19 surveillance variables of the municipalities in which the PHCF were located, using the Mann–Whitney test.

All statistical analyzes were performed using *Stata – version 14 (StataCorp LLC.)*, for complex samples.

The study was approved by the Research Ethics Committee of FSP/USP under CAAE number 31414420.8.0000.5421 and statement number 4,827,811 of July 5, 2021.

## Results

A total of 907 PHCF participated in the survey, corresponding to 95.8% of the selected PHCF, with no differences between the regions. Most respondents were nurses (82.9%), followed by physicians (7.8%). Reflecting the preponderance of FHS in the country, 92.7% of the responses were from PHCF with FHS, with a greater number of PHCF without FHS in the Southeast and South regions (Table 2).

**Table 2:** Characteristics of survey respondents and of the primary health care facilities, July 2021, Brazil.

Brazil and regions^a^	North % (CI 95%)	Northeast % (CI 95%)	Southeast % (CI 95%)	South % (CI 95%)	Midwest % (CI 95%)	Brazil %^b^ (CI 95%)
Respondent’s profession, *p* = 0.0401						
Nurse	79.20	86.28	80.24	83.33	85.25	83.26
	(71.15–85.46)	(81.13–90.2)	(74.8–84.75)	(77.25–88.04)	(77.75–90.53)	(80.53–85.67)
Physician	11.20	7.08	5.65	8.07	9.84	7.33
	(6.73–18.07)	(4.38–11.26)	(3.37–9.32)	(4.91–12.97)	(5.65–16.57)	(5.74–9.31)
Other^c^	9.60	6.64	14.11	8.60	4.92	9.42
	(5.52–16.19)	(4.03–10.73)	(10.30–19.04)	(5.33–13.60)	(2.22–10.56)	(7.59–11.63)
Percentage of respondents with management post or function at the PHCF *p* = 0.3360	59.20	63.27	68.15	62.37	59.02	64.02
	(50.34–67.50)	(56.77–69.33)	(62.07–73.66)	(55.16–69.06)	(50.05–67.42)	(60.63–67.28)
Percentage of PHCF according to the number of family health strategy teams *p* < 0.0001						
0	1.60	1.77	14.92	11.29	2.46	7.35
	(0.4–6.21)	(0.66–4.63)	(10.99–19.93)	(7.46–16.72)	(0.79–7.39)	(5.8–9.25)
1	58.40	71.24	57.26	56.99	59.84	62.78
	(49.54–66.75)	(64.98–76.78)	(51.00–63.29)	(49.75–63.94)	(50.87–68.19)	(59.43–66.02)
2	20.80	10.18	10.48	16.13	17.21	12.57
	(14.54–28.85)	(6.85–14.87)	(7.23–14.97)	(11.5–22.16)	(11.47–25.01)	(10.51–14.96)
3	11.20	5.75	5.65	9.14	10.66	7.04
	(6.73–18.07)	(3.36–9.67)	(3.37–9.32)	(5.75–14.23)	(6.27–17.54)	(5.52–8.95)
4 or more	8.00	11.06	11.69	6.45	9.84	10.26
	(4.34–14.27)	(7.57–15.88)	(8.24–16.34)	(3.69–11.04)	(5.65–16.57)	(8.3–12.61)
Number of consultation offices per PHCF *p* < 0.0001						
2 or less	47.20	50.88	34.27	32.26	40.98	41.87
	(38.58–55.99)	(44.37–57.37)	(28.61–40.42)	(25.91–39.33)	(32.58–49.95)	(38.51–45.31)
3 or more	52.80	49.12	65.73	67.74	59.02	58.13
	(44.01–61.42)	(42.63–55.63)	(59.58–71.39)	(60.67–74.09)	(50.05–67.42)	(54.69–61.49)
Conectivity of the PHCF						
Fixed line *p* < 0.0001	18.40	15.04	79.03	93.01	62.30	50.43
	(12.52–26.22)	(10.94–20.34)	(73.5–83.67)	(88.31–95.91)	(53.34–70.48)	(47.83–53.04)
Cell phones *p* = 0.0002	26.40	20.35	29.03	41.40	35.25	27.77
	(19.39–34.85)	(15.59–26.13)	(23.7–35.01)	(34.51–48.64)	(27.26–44.16)	(24.86–30.88)
Internet connection *p* < 0.0001	76.80	90.71	97.58	98.39	97.54	93.33
	(68.56–83.40)	(86.15–93.87)	(94.71–98.91)	(95.1–99.48)	(92.61–99.21)	(91.4–94.86)
Good quality internet and adequacy to the activities of the PHCF *p* < 0.0001	58.40	78.32	77.42	81.72	71.31	76.26
	(49.54–66.75)	(72.45–83.23)	(71.78–82.21)	(75.48–86.66)	(62.62–78.67)	(73.23–79.05)
PHCF availability of computers with camera, microphone and internet connectivity *p* = 0.0066	16.80	26.99	25.81	38.71	27.87	27.46
	(11.19–24.44)	(21.59–33.17)	(20.73–31.63)	(31.96–45.93)	(20.60–36.52)	(24.49–30.65)
Use of private cellphone to contact health care users *p* < 0.0001						
No	7.20	4.87	8.87	14.52	3.28	7.58
	(3.78–13.30)	(2.71–8.59)	(5.90–13.12)	(10.13–20.37)	(1.23–8.45)	(5.99–9.55)
Yes–eventually	26.40	22.57	37.90	30.65	26.23	29.21
	(19.39–34.85)	(17.57–28.50)	(32.06–44.12)	(24.42–37.67)	(19.16–34.79)	(26.20–32.42)
Yes–frequently	66.40	72.57	53.23	54.84	70.49	63.21
	(57.64–74.16)	(66.36–78.01)	(46.98–59.37)	(47.61–61.87)	(61.77–77.94)	(59.88–66.42)
Remote modalities COVID-19 treatment						
Phone calls *p* < 0,0001	69.60	80.97	89.52	89.25	90.98	84.68
	(60.95–77.06)	(75.3–85.59)	(85.03–92.77)	(83.90–92.97)	(84.41–94.95)	(82.01–87.02)
WhatsApp messages *p* < 0,0001	65.60	73.01	56.05	70.97	65.57	66.09
	(56.82–73.43)	(66.83–78.41)	(49.79–62.12)	(64.01–77.06)	(56.68–73.49)	(62.78–69.26)
Video calls *p* = 0,0075	19.20	26.99	16.94	17.20	16.39	20.91
	(13.19–27.10)	(21.59–33.17)	(12.75–22.14)	(12.42–23.35)	(10.80–24.10)	(18.19–23.92)
Remote modalities continuity of routine care						
Phone calls *p* = 0,0001	33.60	34.51	50.40	49.46	49.46	42.63
	(25.84–42.36)	(28.58–40.97)	(44.19–56.61)	(42.31–56.64)	(42.31–56.64)	(39.29–46.05)
WhatsApp messages *p* = 0.8994	42.40	45.13	41.94	42.47	45.08	43.5
	(34.01–51.26)	(38.74–51.69)	(35.93–48.19)	(35.54–47.71)	(36.45–54.02)	(40.09–46.98)
Video calls *p* = 0.4336	15.20	15.93	15.32	9.68	16.39	14.83
	(9.89–22.65)	(11.70–21.32)	(11.34–20.38)	(6.17–14.86)	(10.8–24.1)	(12.5–17.5)
COVID-19 treatment for users with severe conditions *p* < 0.0001	37.60	19.91	29.84	47.85	34.43	29.66
	(29.52–46.44)	(15.19–25.65)	(24.45–35.85)	(40.74–55.05)	(26.51–43.32)	(26.72–32.78)
PHCF organized a unique workflow for users with respiratory symptoms in 2020 *p* < 0.0001	81.60	85.84	94.76	93.55	89.34	89.69
	(73.78–87.48)	(80.64–89.82)	(91.17–96.94)	(88.96–96.31)	(82.46–93.73)	(87.36–91.62)

^a^Number of responses: Brazil = 907; North = 125; Northeast = 226; Southeast = 248; South = 186; Midwest = 122.^b^Result for Brazil calculated according to sample weights;^c^Dental Surgeon, Physiotherapist, Social assistant, Physical educator, Nutritionist, Pharmacist, Psychologist;* *p* values Chi-square tests.

The majority of respondents, 62.8% (59.4–66.0), work in PHCF with only one FHS team and with an equally small availability of consultation offices. The PHCF’s connectivity structure shows marked differences between the Brazilian regions (Table 2). The index values of the four analyzed axes, for the country as a whole, did not exceed 70, with the highest value in the surveillance axis and the lowest in the social support axis (Table 3).

**Table 3:** COVID-19 Primary Healthcare Index-CPI axes, dimensions and aggregate variables: average scores and 95% CI. Brazil and regions, 2021.

CPI axes and aggregate variables	North	Northeast	Southeast	South	Midwest	Brazil	Significant differences*
1. COVID-19 TREATMENT	65 (61–68)	64 (62–67)	68 (65–70)	70 (67–73)	64 (61–67)	66 (65–67)	S > N; NE
1.1 Equipment (oximeter, O_2_, thermometer)	58 (53–63)	55 (52–59)	74 (70–77)	84 (81–87)	71 (66–76)	67 (65–69)	S > all regions SE; MW > N;NE
1.2 Supplies for RT-PCR and rapid tests (Ag)	45 (38–53)	50 (44–55)	54 (49–59)	63 (57–69)	48 (40–55)	52 (49–55)	S > N; NE;MW
1.3 Change in PHCF (PHC facilities) operation	54 (48–60)	60 (57–64)	64 (61–67)	66 (63–70)	63 (58–67)	62 (60–64)	S; SE > N
1.4 Institutional transportation/referrals	93 (91–95)	94 (93–96)	95 (94–97)	94 (92–95)	92 (90–95)	94 (93–95)	ns
1.5 Remote follow-up modalities	52 (46–57)	60 (56–64)	54 (51–58)	59 (55–63)	58 (53–63)	57 (55–59)	NE > N; SE
1.6 In-person consultations	74 (66–81)	60 (54–67)	67 (61–72)	63 (56–70)	62 (53–70)	64 (61–67)	ns
1.7 HV (home visit) or peridomiciliary visit	77 (69–84)	70 (64–76)	67 (61–72)	59 (52–66)	58 (49–67)	67 (64–70)	N > S; MW
2. HEALTH SURVEILLANCE	69 (65–73)	71 (69–74)	71 (68–74)	69 (65–72)	67 (64–71)	70 (69–72)	ns
2.1 Territory Information	63 (56–71)	76 (71–81)	74 (70–79)	70 (64–76)	66 (58–73)	73 (70–75)	NE > N; MW
2.1 ILI (Influenza-like Illness) Notification	77 (69–84)	72 (66–78)	72 (66–77)	60 (53–67)	65 (56–73)	70 (67–73)	S < N. NE; SE
2.3 Test collection	26 (19–32)	21 (16–26)	36 (31–41)	44 (38–51)	32 (25–39)	30 (28–33)	S > N; NE < S; SE
2.4 Health Education	95 (92–98)	97 (96–99)	89 (86–92)	89 (86–92)	91 (87–95)	93 (92–94)	N > S; SE; NE > S; SE;MW
2.5 Case Surveillance	85 (80–90)	90 (87–93)	83 (79–87)	80 (75–84)	83 (78–89)	86 (84–87)	NE > S
3. SOCIAL SUPPORT	60 (56–64)	62 (60–65)	57 (54–60)	57 (53–60)	61 (57–64)	59 (58–61)	NE > S; SE
3.1 PHCF and CHW social support activities	61 (56–66)	66 (63–69)	57 (53–60)	57 (53–61)	66 (61–71)	61 (60–63)	NE; MW > S;SE
3.2 Population initiatives (knowledge of the PHCF)	81 (75–86)	84 (80–87)	78 (74–82)	77 (72–81)	82 (76–87)	80 (78–83)	ns
3.3 Social articulation	37 (30–44)	37 (32–42)	35 (30–40)	37 (31–42)	34 (27–41)	36 (33–39)	ns
4. ROUTINE CARE	66 (64–69)	66 (64–68)	60 (58–62)	61 (59–64)	64 (61–67)	63 (62–65)	N; NE > S; SE
4.1 Routine consultations/care	82 (78–85)	81 (78–84)	74 (71–77)	80 (77–83)	81 (77–85)	79 (77–80)	SE < all regions
4.2 Maternal-child health care	86 (82–90)	88 (85–90)	83 (80–86)	83 (80–87)	86 (83–90)	85 (84–87)	ns
4.3 Routine vaccination	90 (86–95)	96 (94–98)	88 (84–91)	86 (81–90)	88 (83–93)	91 (89–93)	NE > all regions
4.4 NTCD care	89 (86–93)	86 (82–89)	82 (78–85)	83 (79–87)	85 (80–89)	84 (82–86)	ns
4.5 Home care	77 (71–83)	73 (68–78)	62 (57–67)	74 (68–79)	69 (63–75)	70 (67–72)	SE < N; NE; S
4.6 Multidisciplinary care–(NASF/FHSC)	48 (40–56)	47 (41–52)	33 (28–39)	30 (24–36)	48 (40–56)	40 (37–43)	N; NE > S; SE; MW > SE
4.7 Mental Health Care	82 (76–88)	80 (76–85)	75 (70–79)	78 (73–83)	79 (73–85)	78 (76–81)	ns
4.8 Modalities of remote patient follow-up	41 (37–45)	42 (40–45)	43 (40–45)	41 (38–44)	43 (40–47)	34 (31–36)	ns
4.9 List of priority patients	90 (84–95)	96 (93–98)	92 (89–96)	93 (89–96)	91 (86–96)	93 (92–95)	ns
4.10 Electronic request of exams/Prescription	9 (4–14)	7 (4–10)	10 (6–13)	9 (6–13)	13 (8–18)	9 (7–11)	MW > NE
4.11 Home delivery of medication	43 (34–52)	42 (36–49)	30 (24–36)	25 (19–31)	31 (23–39)	35 (32–38)	N; NE > S; SE
INDEX (CPI)	65 (63–67)	66 (64–68)	64 (62–66)	64 (62–66)	64 (62–67)	65 (64–66)	ns
Collective dimension	64 (61–67)	66 (64–68)	62 (60–64)	60 (58–62)	62 (59–65)	63 (62–65)	NE > S
Individual dimension	65 (63–68)	64 (62–66)	65 (63–67)	69 (67–71)	66 (63–69)	65 (64–66)	S > NE

*Dunn test *p* < 0.05; ns–not significant.

In the ‘COVID-19 treatment’ axis, we found the lowest values for the availability of tests and remote modalities for monitoring cases. Oppositely, institutional transport and referral of critically ill patients exhibited higher scores. A greater completeness of actions is observed in the PHCF in the South region when compared to those in the North/Northeast. This performance pattern stems from greater availability of supplies and tests, as well as changes in the opening hours of the units and the specific workflows created for the respiratory symptomatic patients. Contrarily, the introduction of modalities such as remote monitoring was greater in the Northeast/North regions (Table 3).

The Surveillance axis attained the highest value and was also the most homogeneous one, with no differences between regions. Health education activities, which include encouraging social isolation and other preventive initiatives were carried out to a high degree and in practically all PHCF in the Northeast/North regions. The consequences of low scores in test collection in the PHCF may have been partly minimized by a higher value related to the information on confirmed cases and hospitalizations of residents living in the PHCF coverage area.

In the Social Support axis, we observed lower values in the articulation of the PHCF with other social sectors and with organized community movements. However, important activities stand out, especially in the northeast region, such as enrollment in cash transfer programs and/or basic food basket distribution led by PHCF, together with CHW (Table 3).

The Routine Care axis attained an average value, with emphasis on the Northeast/North regions. The actions with the lowest values were electronic prescriptions and requests for exams, and inclusion of remote patient monitoring modalities. Oppositely, traditional actions such as routine vaccination, chronic diseases, and maternal and child health care showed high values, with no differences between regions, except for routine vaccination, which was higher in the Northeast (Table 3).

The individual dimension of care returned a greater value in the South region units when compared to those of the Northeast, and the relationship is exactly the opposite in the Collective dimension, with the Northeast exhibiting higher scores than the South, with the Midwest showing an intermediate pattern (Table 3).

We contrasted the PHCF above and below the median CPI with socioeconomic, political, and health indicators, and we found an association between those with the highest CPI and their location in municipalities with lower results on HDIM, *per capita* GDP, population, percentage of votes on President Bolsonaro (2018), number of hospitals, and ICU beds. Reversely, higher FHS coverage was associated with a higher CPI (Table 4). Regarding the classification of the municipality as rural or urban, the difference in the proportions of above median CPI was significant, as 66% of the rural PHCF had better index results, whereas in urban areas, the percentage was 43%. We did not observe any differences regarding the mortality rate or the number of confirmed cases/100,000 inhabitants.

**Table 4:** Socioeconomic, demographic, political, structural and health surveillance variables: median and interquartile range (IQR) distributions and associations with COVID-19 Primary Healthcare Index-CPI.

Variable	CPI below median score (≤65)	CPI above median score (>65)	Significant associations*
	Median (IQR)	Median (IQR)	*p*
HDIM^1^	72 (65–76)	70 (62–75)	0.0135*
HDIM–education	64 (55–70)	61 (52–68)	0.0103*
HDIM–life expectancy	83 (79–85)	82 (79–85)	0.0964
HDIM–income	71 (63–75)	69 (60–74)	0.0163*
GDP ~ ^2^ (R$)	1,560,979 (468,057-8,165,793)	636,170 (178,059-4,040,635)	<0.0001*
GDP per capita (R$)	25,963 (15,884-40,668)	22,671 (13,025-38,369)	0.0144*
Population estimates^3^	62,508 (23,331-232,491)	30,430 (13,491-126,970)	<0.0001*
% of voting in Bolsonaro^4^	63 (38–72)	57 (33–69)	0.0028*
Hospitals and equipment^5^			
Hospitals (*n*)	2 (1–7)	1 (1–4)	<0.0001*
Ventilators (*n*)	15 (2–122)	5 (0–56)	<0.0001*
Hospitals with ICU^5^			
ICU–types			
ICU ii Adult (*n*)	0 (0–1)	0 (0–1)	0.0063*
ICU ii Pediatric (*n*)	0 (0–0)	0 (0–0)	0.2171
ICU ii Adult Covid (*n*)	0 (0–1)	0 (0–1)	0.0001*
ICU ii Pediatric Covid (*n*)	0 (0–0)	0 (0–0)	0.4688
Total of ICU (*n*)	1 (0–4)	0 (0–2)	0.0001*
ICU beds^5^			
ICU beds ii Adult (*n*)	0 (0–19)	0 (0–10)	0.0070*
ICU beds ii Pediatric (*n*)	0 (0–0)	0 (0–0)	0.2177
ICU beds ii Adult Covid (*n*)	0 (0–20)	0 (0–12)	0.0003*
ICU beds ii Pediatric Covid (*n*)	0 (0–0)	0 (0–0)	0.4723
Total of ICU beds (*n*)	8 (0–53)	0 (0–30)	0.0001*
Coverage^6^			
Family Health Strategy (%)	80 (50–100)	97 (65–100)	<0.0001*
Primary Health Care (%)	90 (62–100)	100 (77–100)	<0.0001*
COVID-19–cases and deaths^7^			
Confirmed cases/100 k	10,815 (7,330-14,136)	10,376 (6,903–14.190)	0.1946
Mortality rate	0.024 (0.018–0.032)	0.024 (0.017–0.034)	0.5553

*Mann–Whitney significant test results *p* < 0.05.^1^UNDP–United Nations Development; ^2^IBGE–Brazilian Institute of Geography and Statistics; ^3^TCU–Federal Accounting Court; ^4^TSE–Superior Electoral Court; ^5^CNES–National Register of Health Facilities ^6^e-gestor AB–Primary Care Information System Program; ^7^Wesley Cota repository: wcota/covid19br. Structural and health surveillance variables: median and interquartile range (IQR) distributions and associations with COVID-19 Primary Healthcare Index-CPI.

## Discussion

Brazil’s pre-existing conditions of great social inequality and high informal employment, like most countries in Latin America (24, 25), demand a greater role from PHC in guaranteeing care, especially for the most vulnerable populations, which requires the performance of social and intersectoral support actions.

This study shows that the most vulnerable localities, despite their structural conditions, had a more complete set of PHC initiatives, especially in the collective dimension, of surveillance and social support (North/Northeast regions). These localities presented a high coverage of the Family Health Strategy, which prevented worse outcomes, such as higher COVID-19 mortality rates. In contrast, the South/Southeast regions scored better on the individual dimension, especially in COVID-19 treatment, and had fewer community attributes, which ultimately balanced the scales, with no significant differences in COVID-19 mortality rates.

Although working conditions have significantly improved in recent years, with several programs improving the PHCF structure and HR provision, the lack of structure remains a reality in the country, especially in the North/Northeast regions (26, 27), which concentrate more vulnerable populations, with less access to a structured network of health services, once again reissuing the Inverse Care Law (28).

The physical structure, represented by the number of available consultation offices, was an obstacle to the offer of adequate care during the pandemic when different workflows must be established for symptomatic respiratory patients. That was overcome by the teams’ creativity, with exclusive flows in outside areas of the PHCF. Despite the structural difficulties, a rapid and important change was implemented in the organization of both internal and external flows, i.e., what was within the governance of the PHC teams, was carried out promptly. This rapid reorganization of PHCF was also observed in other countries (21), being one of the most common responses in these services.

The lack of effective connectivity in all Brazilian PHCF was drastically felt at this time, especially in the Northeast/North regions. Even without institutional resources, the committed PHC professionals started using their equipment, their personal mobile phones, to guarantee care. This contributed to a rapid change in the care processes, with the introduction of different forms of remote monitoring, both for COVID-19 and routine care. A more effective national telehealth policy, with adequate financial support, such as what happened in Australia (29), would have resulted in scaling and would have made a difference in the Brazilian scenario.

The introduction of remote modalities to monitor users with COVID-19 was a worldwide practice in PHC, having been reported in the most diverse realities (30). Baines et al. carried out, before the pandemic, a scoping review on the obstacles and facilitators of the introduction of remote actions in PHC scenarios (31). Among the obstacles identified, are the lack of understanding of the purposes and effectiveness of remote consultations, the conception that only part of the users would be able to adhere to this practice, and that it should be aimed only at less complex cases. Within a few months, these issues were minimized for most PHC professionals worldwide, who were rapidly forced to incorporate these tools into their daily lives. Although the changes are extensive, the implementation of remote procedures is easier in larger health units, with good administrative and information technology support, a reality far removed from that observed in Brazil (32).

One of the greatest difficulties faced by PHC was, undoubtedly, to guarantee the continuity of care, which did not occur only in Brazil. Especially at the beginning of the pandemic, the focus of PHC performance was largely on the screening and care of patients with respiratory conditions (18, 30, 33). At first, elective care was significantly reduced in almost all countries; however, this changed with the progression of the pandemic. The results found herein are in line with the world scenario but already indicate adaptations in the work processes to maintain routine care, especially for priority groups. In order to offer continuous and quality health care of the Brazilian PHC, it is crucial to improve the team capacity, their articulation with the other levels of the system, the coordination of care, and the infrastructure. These improvements are essential, as it is very likely that PHC services will experience an increase in routine care demand, especially with higher levels in the prevalence of mental health disorders and of long-term COVID-19 (34, 35).

Haldane et al. (36) analyzed the first governments’ proposals in 14 countries on PHC services’ performance, using Patel‘s framework (37), specially designed for assessing PHC performance, in pandemic contexts. Overall, the documents proposed changes in the work processes, suggesting procedures that would guarantee continuity of care and reduce the risk of contagion for users and professionals. However, few of them emphasized surveillance actions.

The need to include surveillance actions linked to PHC services stands out in the international literature (38), although the PHC model most often incorporated in the central capitalist countries’ health systems is very focused on the physician, the General Practitioner, with a weak community dimension and scarce territory surveillance actions. From this perspective, the Brazilian FHS model would start from a higher level in the fight against COVID-19, even more so when it had a network of more than 43,000 FHS teams and 300,000 CHWs. Unfortunately, this potential was wasted by the policy carried out by the Ministry of Health, (39).

The PHC teams carried out the surveillance actions under their governance, but the lack of tests prevented the implementation of the test, trace, and isolation model to contain the pandemic. This model, associated with social isolation measures, had positive results in some countries (40) and was identified as one of the most important pillars to combat the spread of the pandemic, before the availability of vaccines. And it could have lessened the immense impact of COVID-19 in Brazil.

Brazil comprises, one could say, many ‘countries’, as it is characterized by immense social inequalities, demanding a greater role from PHC in guaranteeing care, especially for the most vulnerable populations, which requires the performance of social and intersectoral support actions. Despite its importance, this axis was the most fragile one, especially in carrying out intersectoral actions, demonstrating the immense challenge to incorporate these practices into the daily life of PHC teams. The exception was observed in the Northeast region, where social support actions were carried out in more than half of the PHCF.

The results also indicate different regional profiles in the fight against COVID-19. Overall, greater completeness was observed for collective actions in the Northeast/North regions, conveying their greater adherence to the community approach. This approach demands changes in the work processes, supported by the FHS’ multiprofessional characteristic and CHW’s central presence. Reversely, the individual dimension is evident, especially in the PHCF of the South/Southeast regions. In part, the greatest performance difficulties in the individual dimension in the North/Northeast regions originate from the lack of structural conditions.

In recent years, the “*Mais Médicos*” (More Doctors) Program, which brought physicians to remote and disadvantaged areas, was discontinued, leaving hundreds of municipalities without doctors. Changes in federal funding and initiatives toward promoting an individual assistance model constrain the universality of care. With a distinct pace of implementation in the country, these setbacks produce a greater diversification of care models (41), as has been seen in our study. When we change the scale of analysis, leaving that of the regions and moving on to the municipalities, the results found also show the potential and limits of PHC. The CPI was higher precisely in the PHCF located in municipalities that would have, to begin with, greater difficulty in autonomously coping with COVID-19, which certainly contributed to the reduction of health inequities.

In this sense, the expanded role of the PHCF and its plasticity is reinforced, including when it provides care for users with severe conditions. This scenario would be even more virtuous if the effective integration of PHCF with a network of services with other levels of complexity were guaranteed throughout the country.

### Strengths and limitations

The major strength of our study is its pioneer timing and setting, as it was the first national study carried out in Brazil with a representative sample of PHCF. At the beginning of the pandemic, we conducted a first survey, but with a convenience sample (42). Thus, the present study is a portrayal of the reorganization of the Brazilian PHC after 18 months of the pandemic and of several disputes on the Brazilian health policy during the period.

The main limitation of this study is the comparison of municipal-level variables with the index CPI scores, which were built in a macro-regional logic. These associations should be analyzed with caution, but they reflect different realities at the regional level as well, which would be much more aggregated if regional indicators were employed.

This study employed a cross-sectional design, which was adequate to answer the research questions during the pandemic period of July–November 2021, It does not involve causality issues, but also includes limitations, as one cannot follow up the organizational changes and processes afterward.

It’s imperative to set the FHS once again as the priority comprehensive health care model for PHC. Strengthening community attributes has a huge impact on service delivery and PHC outputs, and will most certainly affect health outcomes as well, especially if balanced with adequate structural resources, which should be further studied in the future.

## Conclusion

The study results showed the power of the PHC to face the pandemic, given the size of the network of family health teams and the diversity of agents that constitute it to perform their health surveillance and comprehensive and universal care functions.

Although it reduced the volume of routine activities, PHC reinvented itself and faced a scenario of strong structural needs, often maximizing public resources by combining them with private ones, demonstrating its strong commitment to population health.

It is essential to further activate the FHS’ community attributes for controlling the pandemic; strengthening the integration of the SUS services network and the association with solidarity initiatives of community organizations and intersectoral coordination to support the population in its numerous vulnerabilities.

The Brazilian FHS project and the SUS project are crucial for the construction of an egalitarian, democratic, and humanely developed society, capable of facing not only the COVID-19 pandemic but also the other challenges of the Brazilian health context, with protagonism and resilience.

## Data availability statement

The raw data supporting the conclusions of this article will be made available by the authors, without undue reservation.

## Ethics statement

The studies involving humans were approved by the research was approved by the Ethics and Research Committee of the School of Public Health. University of São Paulo (FSP/USP) under reference number CAAE 31414420.8.0000.5421 and statement number 4.827.811 as of July 5th 2021. The studies were conducted in accordance with the local legislation and institutional requirements. The participants provided their written informed consent to participate in this study.

## Author contributions

AB contributed to the conception and design of the work, analysis, interpretation of data, and having drafted the work and revised it. LG contributed to the conception and design of the work, analysis, and interpretation of data and revision. PM contributed to the conception and design of the work, analysis, and interpretation of data and revision. SS contributed to the analysis, interpretation of data, having substantively revised the work. LF, MM, FN, and GC contributed to the conception and design of the work, analysis, and interpretation of data and revision. PC contributed to the analysis and interpretation of data and revision. MA contributed to the design of the work and revision. All authors contributed to the article and approved the submitted version.
