# Supplementary material for: The Brazilian primary health care response to the COVID-19 pandemic: individual and collective approaches
Source: Front Public Health. 2023 Dec 8;11:1212584. doi: 10.3389/fpubh.2023.1212584 (PMC10748390; doi:10.3389/fpubh.2023.1212584)
Supplement: Supplementary file 1 [file Table_1.docx]

**Supplementary Material: Index composition**

| **Table of Contents** | **page** |
| --- | --- |
| Presentation | 2 |
| 1. Correlations between the index, the axes and its variables | 2 |
| 2. Factor and consistency analysis | 3 |
| References | 8 |
| Table 1 - Correlations (Spearman) between variables, axes and the index (CPI) | 3 |
| Table 2 – Results of factor analysis; principal components and *oblimin* rotation (p<0.0001): with three factors | 4 |
| Table 3 - Internal consistency analysis, comparison before and after factor analysis (FA) and changes in variables between the axes according to the theoretical framework | 5 |
| Figure 1 – Axes, variables and collective and individual dimensions of the Index (CPI) | 6 |
| Graph 1 - Distribution of individual and collective dimensions to face the Covid-19 pandemic in PHC in the Brazilian regions (test after factor analysis) | 7 |
| Chart 1 – CPI axes, with aggregate variables, selected components and its assigned values | 9 |

In this supplement, we will detail the steps of the index composition (Covid-19 PHC Index -CPI), so that readers can consult the step-by-step of its configuration. The analysis of correlations between the index, the axes and their respective variables will be presented, as well as the factor and consistency analysis.

After consulting the research group, we carried out a few rounds to redefine the best way to structure the variables, which were constructed in an aggregate or individual way, according to the theoretical frameworks, the underlying concepts, the current topic and data from the research. The final configuration (**Chart 1**) displays 59 component variables, aggregated into 26 variables consisting of four axes: Covid-19 Treatment, Health Surveillance, Social Support and Continuity of Care.

**1. Correlations between the index, the axes and its variables**

To evaluate the correlations between the composite index, the axes and the variables, we performed Spearman’s correlation tests (*rho*) between the axes or between them and the variables and we also tested the variables among themselves, inside and outside the axes (Miot, 2018). The correlation between the index and the axes was stronger for Health Surveillance and Covid-19 Treatment, followed by Social Support, being only moderate for the Continuity of Care axis (**Table 1**). The variables that stood out the most, in the correlation with the index, were those related to Covid-19 detection tests (RT-PCR and Ag); to the modalities of remote patient follow-up, home visits, case surveillance and social articulation. The Continuity of Care axis showed stronger correlations for home care, maternal and child care and mental health care. The strongest correlations between the axes were perceived between Health Surveillance and Covid-19 Treatment (moderate). These occurred mainly due to the variables related to test collection and the sufficient amount of supplies to perform the tests. Within the axes, the strongest correlations were obtained between the variables information on the territory and case surveillance (Health Surveillance); and between those in the block of routine care and consultations (Continuity of Care).

**Table 1** - Correlations (Spearman) between variables, axes and the index (CPI).

| **Axes and variables** | **Covid-19 treatment** | **Health surveillance** | **Social support** | **Continuity of care** | **Index** |
| --- | --- | --- | --- | --- | --- |
| **Axis 1. Covid-19 Treatment** |  | **0.58** | 0.27 | 0.26 | **0.74** |
| 1.1 Equipment (oximeter, O_2_, thermometer) | **0.44** | 0.25 | 0.09 | 0.08 | 0.30 |
| 1.2 Supplies for RT-PCR and rapid tests (Ag) | **0.64** | **0.51** | 0.16 | 0.12 | **0.52** |
| 1.3 Change in PHCF (PHC facilities) operation | 0.15 | 0.29 | 0.14 | 0.14 | 0.36 |
| 1.4 Institutional transportation/referrals | **0.47** | 0.11 | 0.10 | 0.07 | 0.15 |
| 1.5 Remote follow-up modalities | **0.58** | 0.29 | 0.28 | 0.32 | **0.47** |
| 1.6 In-person consultations | **0.64** | 0.33 | 0.06 | 0.06 | 0.38 |
| 1.7 HV (home visit) or peridomiciliary visit | **0.60** | 0.28 | 0.19 | 0.22 | **0.45** |
| **Axis 2. Health Surveillance** | **0.58** |  | 0.32 | 0.22 | **0.77** |
| 2.1 Territory Information | 0.37 | **0.62** | 0.25 | 0.24 | **0.53** |
| 2.1 ILI (Influenza-like Illness) Notification | 0.27 | **0.65** | 0.12 | 0.05 | **0.40** |
| 2.3 Test collection | **0.48** | **0.68** | 0.14 | 0.02 | **0.48** |
| 2.4 Health Education | 0.16 | 0.27 | 0.22 | 0.22 | 0.31 |
| 2.5 Case Surveillance | 0.39 | **0.54** | 0.30 | 0.28 | **0.54** |
| **Axis 3. Social Support** | **0.27** | **0.32** |  | **0.29** | **0.69** |
| 3.1 PHCF and CHW social support activities | 0.19 | 0.20 | **0.63** | 0.25 | **0.46** |
| 3.2 Population initiatives (knowledge of the PHCF) | 0.04 | 0.08 | **0.51** | 0.16 | 0.30 |
| 3.3 Social articulation | 0.27 | 0.31 | **0.77** | 0.18 | **0.56** |
| **Axis 4. Continuity of care** | 0.26 | 0.24 | 0.31 |  | **0.57** |
| 4.1 Routine consultations/care | 0.08 | 0.00 | 0.11 | **0.57** | 0.23 |
| 4.2 Maternal-child health care | 0.01 | 0.01 | 0.07 | **0.59** | 0.21 |
| 4.3 Routine vaccination | 0.04 | 0.06 | 0.06 | 0.33 | 0.16 |
| 4.4 NTCD care | -0.01 | -0.06 | 0.07 | **0.56** | 0.17 |
| 4.5 Home care | 0.14 | 0.09 | 0.16 | **0.64** | 0.32 |
| 4.6 Multidisciplinary care –(NASF/FHSC) | 0.11 | 0.15 | 0.16 | **0.54** | 0.30 |
| 4.7 Mental health care | 0.08 | 0.06 | 0.12 | **0.59** | 0.27 |
| 4.8 Modalities of remote patient follow-up | 0.34 | 0.25 | 0.28 | **0.44** | **0.44** |
| 4.9 List of priority patients | 0.15 | 0.13 | 0.11 | 0.29 | 0.23 |
| 4.10 Electronic request of exams/ Prescription | 0.09 | 0.11 | 0.07 | 0.22 | 0.16 |
| 4.11 Home delivery of medication | 0.23 | 0.19 | 0.24 | **0.49** | 0.39 |

PHCF- - Primary Health Care Facilities; ILI – Influenza-like Illness; CHW – Community Health Worker; NTCD – Non transmissible Chronic Diseases; FHSC – Family Health Support Center

**2. Factor and consistency analysis**

These analyses aim to verify if the set of axes and their variables make sense, i.e., whether their architecture is coherent and consistent (Hodgetts et al., 2006). For that purpose, we performed the Principal Components Analysis, along with orthogonal and oblique rotations. The analysis was highly significant and showed that there should only be three factors or axes, demonstrating the need to readjust the four existing axes. The internal consistency analysis (Cronbach’s alpha) was applied later, to confirm or refute the changes that were made. The axes related to Health Surveillance and Social Support were strongly connected in this analysis (collective dimension), although separated from two of their variables: ILI Notification and Test Collection, which rather identified with the Covid-19 Treatment axis. However, in the latter axis, the transportation/referral variables, remote follow-up and home visits were deemed to be more adequate to the collective factor. Moreover, the Continuity of Care axis also showed variables related to patient follow-up, list of priority patients, home delivery of medications and electronic request of exams and prescriptions, which were also more strongly correlated to the collective dimension. The notification (ILI) remained in the collective dimension, due to the undeniable fact that it belongs to Health Surveillance (**Table 2**).

The data were subsequently confirmed by consistency analysis (Cronbach’s alpha), which successively demonstrated a considerable increase (6 to 31 pp) in internal consistency after these withdrawals (**Table 3**). The values ranged from 39%-70% for the Social Support axis and from 52%-70% for Health Surveillance (both allocated in a single dimension); 55%-64% for Covid-19 Treatment and finally 69%-75% for Continuity of Care. None of the variables showed a substantially higher value than that of its axis, after its removal.

The set of axes remained with internal consistency after the changes (62%). Regarding the set of variables, without considering the axes, the alpha was 77%, not exhibiting considerable increase with the removal of any variable.

**Table 2** – Results of factor analysis; principal components and *oblimin* rotation (p<0.0001): with three factors.

| **Axes and variables** | Factor 1  CC | Factor 2  HS/SS | Factor 3  CT |
| --- | --- | --- | --- |
| Axis 1. Covid-19 Treatment (CT) |  |  |  |
| 1.1 Equipment (oximeter, O_2_, thermometer) |  |  | 0.38 |
| 1.2 Supplies for RT-PCR and rapid tests (Ag) |  |  | 0.71 |
| 1.3 Change in PHCF operation |  |  | 0.30 |
| 1.4 Institutional transport/referrals* |  | 0.14 |  |
| 1.5 Remote follow-up modalities* |  | 0.63 |  |
| 1.6 In-person consultations |  |  | 0.33 |
| 1.7 HV (Home visit) or peridomiciliary visit* |  | 0.36 |  |
| Axis 2. Health Surveillance (HS) |  |  |  |
| 2.1 Information on the Territory |  | 0.48 |  |
| 2.1 IS Notification* |  |  | 0.40 |
| 2.3 Test collection* |  |  | 0.72 |
| 2.4 Health Education |  | 0.32 |  |
| 2.5 Case Surveillance |  | 0.54 |  |
| Axis 3. Social Support (SS) |  |  |  |
| 3.1 PHCF and CHW social support activities |  | 0.39 |  |
| 3.2 Population initiatives (knowledge of PHCF) |  | 0.19 |  |
| 3.3 Social articulation |  | 0.31 |  |
| Axis 4. Continuity of care (CC) |  |  |  |
| 4.1 Routine consultations/service | 0.72 |  |  |
| 4.2 Maternal-child health care | 0.78 |  |  |
| 4.3 Routine vaccination | 0.30 |  |  |
| 4.4 NTCD care | 0.77 |  |  |
| 4.5 Home care | 0.60 |  |  |
| 4.6 Multidisciplinary care (NASF/FHSC) | 0.30 |  |  |
| 4.7 Mental health care | 0.60 |  |  |
| 4.8 Modalities of remote patient follow-up* |  | 0.63 |  |
| 4.9 List of priority patients* |  | 0.26 |  |
| 4.10 Electronic request of exams/prescription* |  | 0.28 |  |
| 4.11 Home delivery of medications* |  | 0.40 |  |

Adequacy of factor analysis to all variables (overall KMO test=0.81);

*variables that would change axis, in relation to the original construct.

CC – Continuity of care; HS – Health surveillance; SS – Social support and – CT Covid-19 Treatment

PHCF- - Primary Health Care Facilities; ILI – Influenza-like Illness; CHW – Community Health Worker; NTCD – Non transmissible Chronic Diseases; FHSC – Family Health Support Center

**Table 3 -** Internal consistency analysis, comparison before and after factor analysis (FA) and changes in variables between the axes according to the theoretical framework.

| **Axes and variables** | **Alpha** | **Alpha** |
| --- | --- | --- |
|  | Before FA | After FA |
| Axis 1. Covid-19 Treatment | 0.55 | 0.64 |
| 1.1 Equipment (oximeter, O_2_, thermometer) | 0.52 | 0.63 |
| 1.2 Supplies for RT-PCR and rapid tests (Ag) | 0.47 | 0.49 |
| 1.3 Change in PHCF operation | 0.50 | 0.63 |
| 1.4 Institutional transportation/referrals | 0.56 | Collective dimension |
| 1.5 Remote follow-up modalities | 0.51 | Collective dimension |
| 1.6 In-person consultations | 0.49 | 0.66 |
| 1.7 HV or peridomiciliary visits | 0.51 | Collective dimension |
| 2.3 Test collection | Axis 2 | 0.66 |
| Axis 2. Health surveillance (Collective dimension=A2+A3*) | 0.52 | 0.70* |
| 2.1 Information on the Territory | 0.45 | 0.67 |
| 2.1 IS Notification | 0.49 | 0.71 |
| 2.3 Test collection | 0.45 | Axis 1 |
| 2.4 Health Education | 0.53 | 0.69 |
| 2.5 Case Surveillance | 0.40 | 0.66 |
| 1.4 Institutional transportation/referrals | Axis 1 | 0.70 |
| 1.5 Remote follow-up modalities | Axis 1 | 0.67 |
| 1.7 Home/peridomiciliary visits | Axis 1 | 0.68 |
| 3.1 PHCF and CHW social support activities | Axis 3 | 0.68 |
| 3.2 Population initiatives (knowledge of the PHCF) | Axis 3 | 0.70 |
| 3.3 Social articulation | Axis 3 | 0.68 |
| 4.8 Patient follow-up modalities | Axis 4 | 0.66 |
| 4.9 List of priority patients | Axis 4 | 0.69 |
| 4.10 Electronic request of exams/ prescription | Axis 4 | 0.70 |
| 4.11 Home delivery of medications | Axis 4 | 0.68 |
| Axis 3. Social Support* | 0.39 | 0.70* |
| 3.1 PHCF and CHW social support activities | 0.22 | Collective dimension |
| 3.2 Population initiatives (PHCF knowledge) | 0.39 | Collective dimension |
| 3.3 Social articulation | 0.30 | Collective dimension |
| Axis 4. Continuity of care | 0.69 | 0.75 |
| 4.1 Routine consultations/service | 0.65 | 0.71 |
| 4.2 Maternal-child health care | 0.65 | 0.69 |
| 4.3 Routine vaccination | 0.69 | 0.76 |
| 4.4 NTCD care | 0.65 | 0.70 |
| 4.5 Home care | 0.64 | 0.71 |
| 4.6 Multidisciplinary care (NASF/FHSC) | 0.67 | 0.77 |
| 4.7 Mental health Care | 0.65 | 0.72 |
| 4.8 Modalities of remote patient follow-up | 0.69 | Collective dimension |
| 4.9 List of priority patients | 0.70 | Collective dimension |
| 4.10 Electronic request of exams /prescription | 0.69 | Collective dimension |
| 4.11 Home delivery of medications | 0.70 | Collective dimension |

Note: In case a single variable exhibits alpha values well above that of its axis, it may not remain in the tested axis. In orange, values close to the cutoff point (axis’ alpha).

* The dimensions of health surveillance and social support were allocated to a collective dimension.

PHCF- - Primary Health Care Facilities; ILI – Influenza-like Illness; CHW – Community Health Worker; NTCD – Non transmissible Chronic Diseases; FHSC – Family Health Support Center

Additionally, **Figure 1** shows the final structure of the variables by axes and dimensions, after carrying out the described steps. The variables in the red boxes have been transferred to another axis/dimension, while the green boxes represent their final position. The variables in the blue boxes have remained on the same axis since their initial formulation. The collective dimension showed a larger set of variables from the individual dimension (n=7), while only the test collection variable took the opposite path.

**Figure 1 –** Axes, variables and collective and individual dimensions of the Index (CPI)

**COLLECTIVE DIMENSION: A2+A3**

Remote follow-up

Patient list

Electronic request for exams and prescription

Home delivery medication

PHCF and CHW activities

PHCF knowledge of population initiatives

Social articulation

**INDIVIDUAL DIMENSION: A1+A4**

Moved away

Included

No changes

**Graph 1**, in turn, allows verifying the gradient observed between the Brazilian regions, corroborating the construction of the individual and collective dimensions, after the reconfiguration of the variables between the axes, according to the factor analysis and theoretical references, combining the Covid-19 Treatment and Continuity of Care (individual dimension) axes and the Health Surveillance and Social Support axes (collective dimension).

**Graph 1** - Distribution of individual and collective dimensions to face the Covid-19 pandemic in PHC in the Brazilian regions (test after factor analysis)

**References**

Hodgetts WE; Hagler P; Hodgetts SP. Exploring the Use of Factor Analysis to Determine the Relevant Dimensions of Outcome for a Given Population in Rehabilitation Science: A Tutorial Journal of Speech-*Language Pathology and Audiology*; 2006; 30(2): 132-141. Available at: <https://cjslpa.ca/files/2006_JSLPA_Vol_30/No_02_81-152/Hodgetts_Hagler_Hodgetts_JSLPA_2006.pdf>

Miot HA. Análise de correlação em estudos clínicos e experimentais. *Jornal Vascular Brasileiro* [online]. 2018, 17(4): 275-279. [Access 7 mar 2021). Available at: <https://doi.org/10.1590/1677-5449.174118>

**Chart 1 – CPI axes, with aggregate variables, selected components and its assigned values**

| **Aggregate variables** | **Component variables** | **Assigned values** |
| --- | --- | --- |
| **Axis 1. Covid-19 Treatment** | | Averages of all component variables, provided they are not missing |
| 1.1 Equipment (oximeter, O_2_, thermometer) | 1.1.1 Oximeter availability,  1.1.2 Availability of O_2_  1.1.3 Availability of infrared thermometer | 0=non-existent  0.5=insufficient  1.0=sufficient |
| 1.2 RT-PCR and rapid (Ag) test supplies | 1.2.1 Access to RT-PCR tests  1.2.1 Access to rapid tests (Ag) |  |
| 1.3 Change in PHCF operation | 1.3.1 Increase in the working hours  1.3.2 Specific flow for respiratory symptoms  1.3.3 Maintenance of flow at the time of research | 0=no; 1;=yes  0=no; 1;=yes  0=no; 1=yes NSA=missing |
| 1.4 Institutional transportation and referral of critically-ill patients | 2.4.1.1 Mobile emergency care service (SAMU)  1.4.1.2 Municipal Department of Health (SMS)  1.4.1.3 Patient’s family transportation  1.4.1,4. Another transportation  1.4.2 Clear definition of patient referral  1.4.3 Care in the referral service | Mean 1.4.1; 1.4.2; 1.4.3  Each variable: 0=no; 1=yes  1.4.1 Aggregate: 0=another transportation or family member; 1= SMS or SAMU  0=no; 1-yes (any municipality)  0=rarely; 0.5=almost always; 1=always; (never referred=NSA |
| 1.5 Remote follow-up modalities | 1.6.1 Phone calls  1.6.2 WhatsApp (messages)  1.6.3 Video calls | 0=no; 1=yes |
| 1.6 In-person consultation | 1.6.1 In-person consultations | 0=no; 1=yes |
| 1.7 Home or peridomiciliary visits | 1.7.1 Home or peridomiciliary visits | 0=no; 1=yes |

PHCF- - Primary Health Care Facilities

**Chart 1 – CPI axes, with aggregate variables, selected components and its assigned values (continued)**

| **Aggregate variables** | **Component variables** | **Assigned values** |
| --- | --- | --- |
| **Axis 2. Health surveillance** | | Averages of all component variables, provided they are not missing |
| 2.1 Territory information | 2.1.1 Information on confirmed cases of covid-19  2.1.2 Information on patients hospitalized due to covid-19 | 0=no; 1=yes  0=no; 1;=yes |
| 2.2 Notification (ILI) | 2.2.1 Influenza-like Illness notification | 0=no; 1;=yes |
| 2.3 Test collection | 2.3.1 Collection of material for RT-PCR tests  2.3.2 Collection of material for rapid test (Ag) | 0=no; 1=yes  0=no; 1;=yes |
| 2.4 Health Education | 2.4.1 Encouraging social isolation  2.4.2 Other preventive initiatives (hand washing, masks and ventilation) | 0=no; 1=yes  0=no; 1=yes |
| 2.5 Case Surveillance | 2.5.1 Case monitoring  2.5.2 Active search for contacts;  2.5.2 Monitoring quarantine/isolation contacts | 0=no; 1=yes  0=no; 1=yes |
| **Axis 3. Social Support** | | Averages of all component variables, provided they are not missing |
| 3.1 Social support activities carried out by the PHCF or the CHW | 3.1.1.1 Distribution of basic food items; or  3..1.1.2 Support of CHW in the distribution of basic food items  3.1.2.1 Access to the *Bolsa Família* (conditional cash transfer program) enrollment; or  3.1.2.2 Support of CHW in the identification of social vulnerabilities | 0=no; 0=performed, but without support from the PHCF; 1=yes (PHCF support)  0=no; 1=yes  0=no; 0=performed, but without support from the PHCF; 1=yes (PHCF support)  0=no; 1;=yes |
| 3.2 Knowledge of the PHCF about the existence of initiatives of the population in its territory | 3.2.1 Improving access to water  3.2.2 Improving the cleanliness of common areas  3.2.3 Distribution of meals  3.2.4 Combating fake news about covid-19  3.2.5 Ensuring conditions for proper isolation | 0=do not know; 1=know (yes or no) |
| 3.3 Social articulation | 3.3.1 Articulation of the PHCF with social movements  3.3.2 Articulation of the PHCF with other sectors (secretariats, companies, churches) | 0=no; 0=do not know; 1=yes |

PHCF- - Primary Health Care Facilities; ILI – Influenza-like Illness; CHW – Community Health Worker.

**Chart 1 – CPI axes, with aggregate variables, selected components and its assigned values (continued)**

| **Aggregate variables** | **Component variables** | **Assigned values** |
| --- | --- | --- |
| **Axis 4. Continuity of care** | | Mean of all component variables, provided they are not missing |
| 4.1 Routine consultations/care | 4.1.1 Offer of spontaneous demand consultations  4.1.2 Offer of medical consultations  4.1.3 Offer of nursing consultations  4.1.4 Offer of dental care. | 0=not performed or suspended; 0.25=adapted with reduced supply; 0.5=adapted without changing supply; 0.75=adapted with increased supply; 1=maintained* |
| 4.2 Maternal-child health care | 4.2.1 Offer of prenatal consultations  4.2.2 Offer of childcare consultations  4.2.3 Offer of preventive examination for Cervical Cancer |  |
| 4.3 Routine vaccination | 4.3.1 Offer of routine vaccination |  |
| 4.4 NTCDs care | 4.4.1 Offer of care to people with SAH, DM and other NTCDs |  |
| 4.5 Home care | 4.5.1 Offer of home care by a higher education professional |  |
| 4.6 Multidisciplinary care (NASF/FHSC) | 4.6.1 Offer of FHSC-PHC Professional Activity |  |
| 4.7 Mental health care | 4.7.1 Offer of mental health care |  |
| 4.8 Remote patient follow-up modalities | 4.8.1 Phone calls  4.8.2 WhatsApp (messages)  4.8.3 Video calls | 0=no; 1=yes |
| 4.9 List of priority patients | 4.9.1 Existence of a list of priority group users | 0=no; 1=yes |
| 4.10 Electronic request of exams/ prescription | 4.10.1 Digital exam request  4.10.2 Electronic prescription | 0=no; 1=yes |
| 4.11 Home delivery of medication | 4.11.1 Existence of home delivery of medication | 0=no; 1=yes |

* This gradient was observed during the analyses in relation to the impact variables: although the adaptations with increased offer are extremely positive when facing the pandemic, they showed slightly lower results than those seen in the situation of maintenance of care, while the other adaptations tended to show less favorable results, especially when there is a reduction in the offer.

NTCD – Non transmissible Chronic Diseases; FHSC – Family Health Support Center
